# Effective buffers in intravenous solutions: reconciling the acid–base effects of organic anions

**DOI:** 10.1186/s13054-026-05857-6

**Published:** 2026-02-20

**Authors:** Micah Liam Arthur Heldeweg, Santje A. S. Slot, Martin Vancura, Martin Krbec, Francesco Zadek, František Duška, Lorenzo Giosa

**Affiliations:** 1 Department of Anesthesiology, Amsterdam University Medical Centers, Boelelaan 1117, 1081HV, Amsterdam, The Netherlands; 2 Department of Anesthesia and Intensive Care Medicine, The Third Faculty of Medicine, Charles University and FNKV University Hospital, Prague, Czech Republic; 3 Department of Intensive Care, OLVG, Amsterdam, The Netherlands; 4 Department of Medicine and Surgery, University of Milan-Bicocca, Monza, Italy

**Keywords:** Acid–base, Buffer, Carbon dioxide, Respiratory, Metabolic, Lactate, Saline, Crystalloid

Dear editor,

We read with great interest the recent publication in *Critical Care* on the metabolic fate and acid–base effects of organic anions used in intravenous solutions [1]. The authors propose that (1) organic anions are buffers that exert direct pH effects, (2) these effects occur independently of their metabolism, and (3) they are best explained by the accompanying sodium load and its impact on the strong ion difference [1, 2]. 

While these statements are not incorrect, they are incomplete and rely on selective use of different acid-base interpretative frameworks. This risks becoming a renewed source of confusion in a field long shaped by disagreement. In fact, these apparently divergent viewpoints represent complementary perspectives on a shared physiological process. Here, we address each of the authors' statements and contribute several physiological and physicochemical aspects that help reconcile the acid-base effects of organic acids in solution.

First, most organic anions do not exert effective buffering in human plasma. From a chemical standpoint, buffering is a molecule’s ability to resist pH changes in a solution via reversible proton binding. The buffering capacity of an organic acid depends on its acid-dissociation constant (pKa) and the pH of the solution. The pKa of monocarboxylate organic acids such as gluconic acid, lactic acid, and acetic acid, is far from the physiological plasma pH range [3]. As a result, in human plasma, they are nearly fully dissociated and uncapable of buffering any acid load (Fig. 1A). The acid–base effect of infusing organic anions into separated human plasma is therefore no different from the effect of infusing strong anions (Fig. 1B). Because they do not act as effective buffers in human plasma, they should be considered strong ions under Stewart’s physicochemical framework and therefore alter strong ion difference [4].

**Fig. 1:**
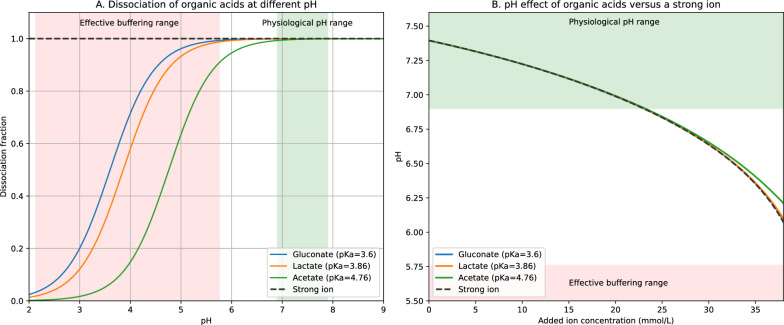
Acid–base contribution of organic acids in physiological pH range. **A**: Dissociation of organic anions at different pH. At a (physiological) pH range of 6.9–7.9, all organic anions remain effectively dissociated, indicating minimal buffering capacity in plasma. **B**: pH effect of organic acids versus a strong ion. Panel B is modelled using the Henderson-Hasselbalch equation. The model assumes a fixed strong ion difference, total weak acid concentration, and partial pressure of CO₂. As the concentration of the organic anions increases, the pH decreases. Over a physiological pH range, the effects of organic acids are consistent with strong ions

Unlike monocarboxylate organic acids, citric acid is a triprotic organic acid; meaning it undergoes three successive dissociation (or proton donation) steps, each characterized by a distinct pKa value. Its third dissociation constant (pKa of 6.4) lies closest to physiological pH, resulting in minimal buffering capacity [3]. However, citrate exists predominantly in its fully deprotonated trivalent anionic form, theoretically contributing approximately 3 mEq of negative charge per mmol infused into plasma. In practice, calcium binding offsets part of this negative charge. While citrate may exert effective buffering effects in fluids with lower pH (e.g. urine), in human plasma, unless metabolized, it is strongly acidifying [5].

Second, acid–base effects of organic anions depends on their removal from plasma. When sodium salts of organic anions are infused, they function as strong cations and anions with a net strong ion difference of zero. If infused into a solution with a positive strong ion difference (e.g. plasma), and in the absence of other changes or removal, the immediate effect is a reduction of its strong ion difference and an acidosis (Fig. 2A). However, organic anions are removed from the extracellular fluid for the purpose of (eventual) intracellular metabolism (Fig. 2B). From a physicochemical perspective, the alkalinizing effect of organic anion infusion can be estimated from the quantity that will be removed without the accompanying strong cation, leading to an increase in strong ion difference (Fig. 2C) [4].

**Fig. 2:**
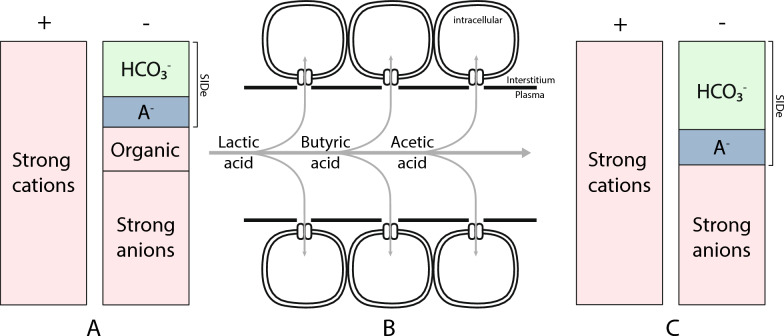
The physicochemical perspective on the infusion of (monocarboxylate) organic acids. **A**: The Gamblegram showcasing components contributing to the final electroneutral state. Organic anions act as strong ions (red), which causes a decreased effective strong ion difference (SIDe) and a metabolic acidosis. **B**: Transfer of monocarboxylate organic acids from the extracellular fluid to the intracellular fluid through the proton-linked monocarboxylate symporter. **C**: The Gamblegram after removal of organic anions, showing an increased SIDe. Moreover, the increase in pH is accompanied by a modest redistribution of strong ions, resulting in a small reduction in strong ion difference (discussed in point 3), and a slight increase in A⁻, thereby causing a small attenuation of the rise in SIDe

From a mechanistic perspective, removal of monocarboxylate organic molecules (e.g. lactate, β-hydroxy butyrate, and acetate) occurs together with a proton through a membrane monocarboxylate transporter [6]. Stoichiometrically, the amount of organic acid removed (millimoles) vastly exceeds the size of the free proton pool (nanomoles). However, as the free proton pool is reduced, buffer-bound protons (e.g., carbonic acid, proteins, phosphates) dissociate back into the pool. Thus, although proton removal is stoichiometrically linked to organic acid removal, the resulting change in free proton concentration is small. This small change shifts the carbonic acid–bicarbonate mass balance equilibrium toward bicarbonate, increasing its relative concentration.

Intracellularly, organic anions converge on pyruvate hydrogenase and the citric acid cycle, where CO_2_ is generated that is subsequently removed by ventilation or hydrated into bicarbonate. Accordingly, infusion of ^13^C-labelled lactate increases the presence of ^13^C-bicarbonate [7]. Therefore, it is true that infusion of metabolizable organic anions eventually gives rise to bicarbonate, as do glucose and other macronutrients. However, it is the initial removal of plasma strong ions that produces the acid–base effect of organic anion infusion, and not the subsequent CO_2_ production, as its total level is tightly controlled by ventilation [8]. Thus, organic anion metabolism itself does not directly produce plasma alkalinization, but it is crucial to maintain the gradient that enables organic anion removal from plasma.

Under normal conditions, the removal of most organic anions for metabolization occurs at high enough rates to support both commonly used descriptions of the alkalinizing effects: a sodium-driven increase in strong ion difference and a concomitant rise in bicarbonate concentration. In contrast, when metabolic capacity is impaired or saturated, organic anion removal is reduced, leading to plasma strong anion accumulation and persistent metabolic acidosis [9]. In the case of gluconate, metabolism is relatively slow, and its clearance from the extracellular fluid occurs via larger, sodium-coupled transport mechanisms. This likely explains its (lower than expected) effect on acid–base balance and its smaller functional contribution to increasing the strong ion difference [10, 11].

Third, observed plasma acid–base alterations cannot be attributed solely to the infused sodium load. While changes in plasma strong ion difference may mostly reflect the infused sodium load when organic acids are fully removed, plasma is not an isolated solution. Rather, it is part of a multicompartment physiological system. Changes in pH elicit buffering responses across multiple compartments (e.g. red blood cells, muscle, bone). As a result, electrolytes are redistributed across compartments to preserve electroneutrality. Numerous clinical and experimental studies have demonstrated that changes in pH are associated with reciprocal changes in strong ion difference, not fully explained by infused electrolyte load itself [12, 13]. These changes are primarily mediated by changes in plasma sodium and chloride concentration. There remains a need for experimental and in vivo studies to fully describe the determinants and magnitude of these responses.

Moreover, infused fluids can influence plasma pH through other determinants than strong ion difference. For example, rapid crystalloid infusion during resuscitation expands intravascular volume and dilutes weak acids (e.g. albumin, phosphate), exerting an alkalinizing effect. This effect is not readily seen with dialysis or slow maintenance fluids and not accounted for by evaluating sodium load alone [14].

Nonetheless, an ion equilibrium–guided approach, i.e. interpreting acid–base effects from measurable ionic charge balance (strong ion difference, weak acids, strong ion gap) across body fluid compartments, certainly offers greater clinical insight into the acid–base effects of organic acids than reliance on inferred metabolic pathways or viewing them solely as bicarbonate precursors.

In summary, most organic anions should be viewed as strong ions in human plasma and influence acid–base balance not through effective buffering, but through their (metabolism-dependent) removal from the extracellular fluid and subsequent increase in plasma strong ion difference and bicarbonate. These processes may be adequately characterized by ion equilibrium parameters, while accounting for dilutional effects of fluid kinetics and multicompartment electrolyte redistribution. A major conceptual advance in understanding the acid–base effects of organic anions lies in recognizing the complementarity of physiological and physicochemical perspectives [15]. Integrating buffering physiology with electrolyte-based plasma chemistry facilitates interpreting complex clinical acid–base disturbances and reconciling the effects of organic anions in intravenous solutions.

## Data Availability

All data used in this study were generated synthetically using Python code; no patient-related data were involved. The code used to generate and analyze the data is available from the authors upon reasonable request.

## References

[1] Ulsamer A, Betbesé AJ, Campos-Gómez A, et al. Buffers in intravenous solutions: is the source of bicarbonate a source of confusion? Crit Care. 2025. 10.1186/s13054-025-05780-2. 41299755 10.1186/s13054-025-05780-2 PMC12750785

[2] Morgan TJ. The ideal crystalloid - what is balanced? Curr Opin Crit Care. 2013;19(4):299–307. 10.1097/MCC.0b013e3283632d46. 23743589 10.1097/MCC.0b013e3283632d46

[3] Goldberg R, Kishore N, Lennen R. Thermodynamic quantities for the ionization reactions of buffers. J Phys Chem Ref Data. 2002;31(2):231–370. 10.1063/1.1416902.

[4] Morgan TJ. The Stewart approach–one clinicians perspective. Clin Biochem Rev. 2009;30(2):41–54. 19565024 PMC2702213

[5] Hamm LL, Simon EE. Roles and mechanisms of urinary buffer excretion. Am J Physiol. 1987. 10.1152/ajprenal.1987.253.4.F595. 3310662 10.1152/ajprenal.1987.253.4.F595

[6] Halestrap AP, Price NT. The proton-linked monocarboxylate transporter (MCT) family: structure, function and regulation. Biochem J. 1999;2:281–99. PMC1220552 10510291

[7] Park JM, Spielman DM, Josan S, Jang T, Merchant M, Hurd RE, et al. Hyperpolarized (13)C-lactate to (13)C-bicarbonate ratio as a biomarker for monitoring the acute response of anti-vascular endothelial growth factor (anti-VEGF) treatment. NMR Biomed. 2016;29(5):650–9. 10.1002/nbm.3509. 26990457 10.1002/nbm.3509 PMC4833516

[8] Heldeweg MLA, Duška F, Krbec M. Understanding buffering of metabolic acidosis in critically ill: keep an open mind. Intensive Care Med. 2025;51(3):650–1. 10.1007/s00134-025-07811-6. 39961839 10.1007/s00134-025-07811-6

[9] Ergin B, Kapucu A, Guerci P, Ince C. The role of bicarbonate precursors in balanced fluids during haemorrhagic shock with and without compromised liver function. Br J Anaesth. 2016;117(4):521–8. 10.1093/bja/aew277. 28077541 10.1093/bja/aew277

[10] Chaussard M, Dépret F, Saint-Aubin O, Benyamina M, Coutrot M, Jully M, et al. Physiological response to fluid resuscitation with ringer lactate versus Plasmalyte in critically ill burn patients. J Appl Physiol. 2020;128(3):709–14. 10.1152/japplphysiol.00859.2019. 32027547 10.1152/japplphysiol.00859.2019

[11] Chowdhury AH, Cox EF, Francis ST, Lobo DN. A randomized, controlled, double-blind crossover study on the effects of 2-L infusions of 0.9% saline and plasma-lyte® 148 on renal blood flow velocity and renal cortical tissue perfusion in healthy volunteers. Ann Surg. 2012;256(1):18–24. 10.1097/SLA.0b013e318256be72. 22580944 10.1097/SLA.0b013e318256be72

[12] Zadek F, Danieli A, Brusatori S, Giosa L, Krbec M, Antolini L, et al. Combining the physical-chemical approach with standard base excess to understand the compensation of respiratory acid-base derangements: an individual participant meta-analysis approach to data from multiple canine and human experiments. Anesthesiology. 2024;140(1):116–25. 10.1097/ALN.0000000000004751. 37616330 10.1097/ALN.0000000000004751

[13] Giosa L, Zadek F, Busana M, De Simone G, Brusatori S, Krbec M, et al. Quantifying ph-induced changes in plasma strong ion difference during experimental acidosis: clinical implications for base excess interpretation. J Appl Physiol. 2024;136(4):966–76. 10.1152/japplphysiol.00917.2023. 38420681 10.1152/japplphysiol.00917.2023 PMC11305652

[14] Carlesso E, Maiocchi G, Tallarini F, Polli F, Valenza F, Cadringher P, et al. The rule regulating pH changes during crystalloid infusion. Intensive Care Med. 2011;37(3):461–8. 10.1007/s00134-010-2095-y. 21152898 10.1007/s00134-010-2095-y

[15] Giosa L, Camporota L, Langer T. On acid-base bilingualism. Intensive Care Med. 2025;51(1):243–4. 10.1007/s00134-024-07734-8. 39661134 10.1007/s00134-024-07734-8

